# Acute Liver Injury Following Outpatient Trimethoprim‐Sulfamethoxazole Use for Small Intestinal Bacterial Overgrowth: A Case Report

**DOI:** 10.1002/ccr3.72989

**Published:** 2026-06-30

**Authors:** Kaylie Duit, Joseph Rattenni, Lauryn Hanson, Aaron R. Kunz

**Affiliations:** ^1^ University of Iowa Iowa City Iowa USA

**Keywords:** chemical and drug induced liver injury, drug‐related side effects adverse reactions, small intestinal bacterial overgrowth, trimethoprim‐sulfamethoxazole drug combination

## Abstract

Drug‐induced liver injury (DILI) is a leading cause of acute liver failure in the United States and remains difficult to predict and diagnose. Trimethoprim‐sulfamethoxazole (TMP‐SMX) is widely prescribed and generally well tolerated, yet rare cases of severe idiosyncratic hepatotoxicity have been reported. We describe a 30‐year‐old woman who developed fever, malaise, leukopenia, and profound hepatocellular injury days after initiating TMP‐SMX and neomycin for small intestinal bacterial overgrowth. TMP‐SMX was discontinued, and treatment with N‐acetylcysteine (NAC) and supportive care was initiated. Liver enzymes, coagulopathy, and renal dysfunction steadily improved, and the patient was discharged on hospital day 6 with complete resolution of laboratory abnormalities within one month. In addition to highlighting another documented case of liver injury caused by TMP‐SMX, this case emphasizes the unpredictable nature of drug‐induced hepatotoxicity, the potential use of NAC in DILI, and reinforces the importance of early recognition, comprehensive evaluation, and prompt withdrawal of the offending agent. Clinicians should maintain vigilance when new constitutional or hepatic symptoms arise during therapy and should report suspected cases to improve collective understanding and patient safety data.

## Key Clinical Message

Clinicians should be aware of the potential for severe liver injury as a result of prescribing trimethoprim‐sulfamethoxazole. Review of reported cases involving trimethoprim‐sulfamethoxazole drug‐induced liver injury trends toward female susceptibility, with diversity in hepatocellular insult. Further studies are needed to explain variability in symptom onset, clinical course, and patient outcomes.

## 1 Introduction

Drug‐induced liver injury (DILI) is the leading cause of acute liver failure in the United States and the most common reason for withdrawal of a medication from the market [1]. While this phenomenon can occur across all classes of medications through a variety of mechanisms, a primary pathway involves the accumulation of toxic metabolites during hepatic metabolism [2]. Other contributing factors include medical co‐morbidities, genetic polymorphisms, and medication‐specific factors such as dose, route of metabolism, and lipophilicity [3]. The most common medications implicated in DILI include antimicrobials and herbal or dietary supplements [4], with antimicrobials possessing a stronger propensity for causing chronic liver injury [5].

In 2022, approximately 2.77 million people in the United States were treated with trimethoprim‐sulfamethoxazole (TMP‐SMX) [6]. It is commonly prescribed for uncomplicated urinary tract infections, certain bacterial respiratory infections (e.g., bronchitis, sinusitis), and skin and soft tissue infections. Given its cost‐effective nature and overall medical utility, the World Health Organization included TMP‐SMX on its list of “essential medicines” [7]. TMP‐SMX has many well‐studied side effects, including photosensitivity, folate deficiency, and acute kidney injury, which all can be biochemically linked to the drug's mechanism of action [8, 9]. Less studied, however, is TMP‐SMX's impact on hepatic function, with one study indicating an incidence rate of 5.1 events per 10,000 person‐years [10]. The pattern of liver injury in TMP‐SMX‐induced liver injury is cholestatic, hepatocellular, or mixed, and while most cases resolve rapidly, the injury may become complicated, prolonged, or fatal [11].

Current evidence suggests that the incidence of DILI is higher in patients who are hospitalized and older in age [3], and persons of female sex or African American race are more likely to develop severe injuries and have poorer outcomes [12]. However, because the clinical presentation of DILI is unpredictable (ranging from asymptomatic elevations in liver enzymes to severe outcomes like jaundice, encephalopathy, and potentially liver failure [1]), accurately studying this occurrence is challenging. In addition, diagnostic criteria for DILI is variable and cases are not routinely reported [10], further complicating the true incidence and ramifications. Given the potential for fatal outcomes, continued reporting and analysis of individual cases, such as this report of TMP‐SMX‐induced liver injury, is crucial for improving understanding, advocating for increased clinical awareness, and preventing adverse patient outcomes.

## 2 Case History/Examination

A 30‐year‐old Caucasian female with a past medical history of attention deficit hyperactivity disorder, migraines with aura, insomnia, seasonal allergies, constipation, and small intestinal bacterial overgrowth (SIBO) presented to a local urgent care clinic with a 1‐day history of fever, malaise, and generalized body aches. Evaluation demonstrated a febrile temperature (39.6°C), with point of care laboratory results significant for elevated transaminases (aspartate aminotransferase [AST] and alanine transaminase [ALT] levels both above the limit of detection for the assay and reported as > 2000 U/L), hyponatremia (Na 133 mEq/L), and leukopenia (WBC 3.0 K/mm^3^). Due to these findings, she was transferred to the emergency department for further evaluation.

Additional history was notable for the patient having started a course of TMP‐SMX 160–800 mg (sig 1 tablet by mouth 2 times daily) and neomycin 500 mg (sig 1 tablet by mouth 2 times daily) for 10 days for SIBO just 8 days prior to presentation to urgent care. She reported taking 10 doses of TMP‐SMX and neomycin before developing fever, malaise, and body aches, and ultimately consumed 13 doses of both medications prior to discontinuation.

The patient had no prior history of elevated liver enzymes or liver disease in her family. She denied smoking, vaping, or illicit drug use, and reported alcohol consumption as two to three beers twice per month. She also denied recent use of acetaminophen or herbal supplements, recent travel, or new environmental exposures preceding symptom onset. Her chronic medications on admission included lisdexamfetamine, zaleplon, and rizatriptan, all taken as prescribed without recent dose adjustments.

Upon evaluation in the emergency department and subsequently on admission to the hospital, the patient noted generalized abdominal pain. Her exam was remarkable for moderate tenderness to palpation in the right upper quadrant. She denied nausea, vomiting, or diarrhea. She was treated for ringworm on her right forearm 5 weeks earlier with topical clotrimazole, and this rash remained faintly visible over the site at time of admission.

## 3 Differential Diagnosis, Investigations, and Treatment

Upon admission, the differential for systemic symptoms combined with profound hepatocellular insult was broad and included numerous infectious, inflammatory, autoimmune, pharmacologic, toxic, and gastroenterological etiologies. The differential diagnoses of highest concern included viral hepatitis, EBV or CMV, autoimmune hepatitis, and drug‐induced injury. Cardiac and vascular etiologies (such as Budd‐Chiari) were also on the differential; however, they were lower in priority and concern.

Admission lab results better quantified the elevated INR, AST, and ALT levels, as well as a lower sodium than the initial urgent care testing (Table 1). A secondary lab workup included the following, all of which were negative: acetaminophen level, HCG, COVID‐19, acute viral hepatitis panel, ceruloplasmin, autoimmune markers (anti‐nuclear antibody, F‐Actin antibody, mitochondrial antibody), HIV, HSV, HAV, HBV, HCV, VZV, CMV, EBV, lactic acid, respiratory pathogen panel, TSH, A1c, phosphatidylethanol, creatinine kinase, troponin, platelet level, and erythrocyte sedimentation rate. Hepatitis E virus serologies were not collected as the patient had no recent exposures or travel to endemic areas. CT imaging of the abdomen in the emergency department was unremarkable, and an echocardiogram obtained 2 days later was normal. A right upper quadrant ultrasound with liver Doppler performed on hospital day 1 demonstrated a mildly fatty liver, but there was no evidence of portal vein thrombosis, portal hypertension, cholelithiasis, choledocholithiasis, or cholecystitis.

**TABLE 1:** Trend of notable lab tests during hospitalization and outpatient follow‐up.

Parameter	Ref range	Day 0	Day 1	Day 2	Day 3	Day 4	Day 5	Day 6	29 days post‐discharge
Sodium (mEq/L)	135–145	125	133	135	134	135	137	138	136
Potassium (mEq/L)	3.5–5.0	3.9	4.2	3.5	3.4	3.3	3.5	3.0	4.0
Calcium (mg/dL)	8.5–10.5	8.5	8.2	8.7	8.5	8.8	8.8	8.4	9.5
Creatinine (mg/dL)	0.51–0.95	0.79	0.69	1.18	1.33	2.79	3.12	3.05	0.63
Albumin (g/dL)	3.4–4.8	4.4	4.6	3.6	3.4	3.3	3.2	3.0	4.6
ALP (U/L)	35–104	72	204	185	210	224	317	351	103
ALT (U/L)	0–35	2766	11,373	8364	6519	4807	3187	2102	32
AST (U/L)	0–35	3106	14,214	10,883	4094	1697	604	213	29
Total bilirubin (mg/dL)	≤ 1.2	0.3	1.8	2.1	3.1	4.0	4.4	3.9	0.6
PT (s)	9–12	17.7	22	20	14	14	13	13	—
INR	0.8–1.2	1.5	2.2	2.0	1.4	1.3	1.3	1.2	—
Lactic acid (mmol/L)	0.5–2.2	1.2	2.4	1.6	1.0	0.7	0.6	0.6	—
Hemoglobin (g/dL)	11.9–15.5	12.7	14.6	12.3	11.0	10.6	9.8	9.6	11.9
Hematocrit (%)	35–47	39	44	37	32	31	28	28	37
WBC (K/mm^3^)	3.7–10.5	3.0	4.5	4.6	4.4	5.3	5.2	4.4	6.8
Platelets (K/mm^3^)	150–400	250	217	191	163	212	213	224	354
MCV (fL)	82–99	82	83	81	80	80	78	78	86
GGT (U/L)	≤ 38	—	68	—	—	—	—	—	—
LDH (U/L)	135–214	—	9534	—	—	—	—	—	—
CRP (mg/dL)	0.0 to < 1.0	2.1	—	—	—	—	—	—	—
Ammonia (μmol/L)	11–51	—	42	—	33	40	43	—	—

**FIGURE 1:**
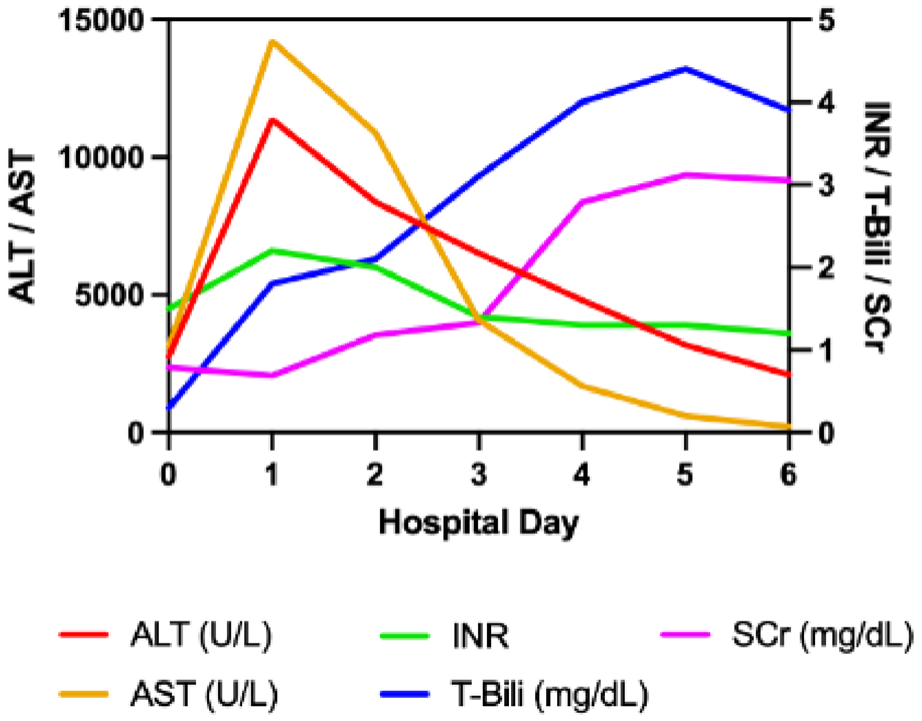
Key laboratory trends throughout hospitalization. ALP, alkaline phosphatase; ALT, alanine aminotransferase; AST, aspartate aminotransferase; CRP, C‐reactive protein; GGT, gamma‐glutamyl transferase; INR, international normalized ratio; LDH, lactate dehydrogenase; MCV, mean corpuscular volume; PT, prothrombin time; SCR, serum creatinine; T‐Bili, total bilirubin; WBC, white blood cells.

On hospital day 1, multiple erythematous, pruritic maculopapular lesions appeared on the extensor surfaces of the bilateral upper and lower extremities, sparing the face, abdomen, and back, though this rash was not observed on subsequent days. The patient also developed mild upper abdominal discomfort, bloating, and nausea. Given symptom evolution in the setting of suspected TMP‐SMX DILI, she was started on an N‐acetylcysteine (NAC) infusion for hepatoprotection. On hospital day 2, her INR continued to increase, and she received a one‐time, 10 mg dose of intravenous vitamin K. By hospital day 3, the patient's abdominal pain and malaise had begun to improve, with all laboratory values continuing to downtrend except for her serum creatinine. This continued to rise over the next few days, eventually peaking at 3.12 mg/dL on hospital day 5. Clinical concern for acute kidney injury occurred approximately 36 hours after contrast exposure. Fractional excretion of sodium and fractional excretion of urea were calculated, resulting in values of 0.5% and 22.9%, respectively, suggesting a contrast‐induced nephropathy versus possible hepatorenal syndrome. She began isotonic bicarbonate therapy on hospital day 4, resulting in gradual improvement of renal function over the subsequent days. Also on hospital day 3, the patient developed a normocytic anemia that persisted through discharge. She was eventually discharged home on hospital day 6 with all laboratory parameters trending toward normal aside from her hemoglobin. The evolution of the patient's key hepatic and renal laboratory markers is shown in Figure 1. The patient never demonstrated any signs of encephalopathy or bleeding during her admission.

## 4 Case Resolution

Follow‐up visits at one day and one week post‐discharge revealed ongoing symptom improvement. Labs obtained 12 days post‐discharge demonstrated a normalized AST (33 U/L) and elevated ALT (118 U/L), with normalization of her creatinine (0.77 mg/dL). At 29 days post‐discharge, her hemoglobin level was reassessed, and it, along with liver and kidney function testing, had normalized.

## 5 Discussion

Because initial signs and symptoms of DILI can be innocuous, a comprehensive evaluation is an essential step in excluding masquerading etiologies and confirming the diagnosis.

The primary objective of this report is to delineate the clinical and laboratory evolution of this injury to highlight the diagnostic challenges and advocate for increased clinical awareness regarding the potentially fatal outcomes associated with a common antibiotic therapy.

In this case, a diagnosis of TMP‐SMX DILI was the leading differential after a thorough diagnostic workup that effectively ruled out alternative causes, such as viral hepatitis, autoimmune hepatitis, metabolic disease, or other hepatotoxic exposures. This diagnosis was also determined given the temporal relationship between starting the medication and symptom onset as well as subsequent improvement in the patient's clinical picture upon discontinuation of TMP‐SMX. Notably, neomycin, the other antibiotic the patient was taking for SIBO, was excluded as a cause of the liver injury due to the lack of reported cases linking it to liver enzyme elevations or symptomatic hepatotoxicity in published literature [13]. Additionally, its limited systemic absorption supports this, as it is unlikely to reach concentrations in the bloodstream that could induce liver damage [13]. Moreover, the patient had taken neomycin in the past with no reported similar presentation while this was her first use of TMP‐SMX. Her hypersensitivity‐like features, including fever and rash, were consistent with reported cases of TMP‐SMX‐induced liver injury that are often mediated by immune mechanisms [11]. When all of the above features and clinical course of our patient were combined into the hepatocellular‐insult algorithm of the Roussel Uclaf Causality Assessment Method (RUCAM) instrument, the patient received a total score of 7, indicating a “probable” cause of drug‐induced liver injury [14].

While this patient shares similar demographic and clinical presentation features with other cases reported in the literature (Table 2), there are several distinctive elements to her case.

**TABLE 2:** Comparison matrix of bactrim DILI cases.

Case report author	Duit et al. (2026)	Onyirimba et al. [15]	Hindosh et al. [16]	Aljaras et al. [17]	Van Asperdt and De Moor [18]	Green et al. [19]	Sharma et al. [20]	Patel et al. [21]	Slim et al. [22]	Abdulhamid and Lehr [23]	Ng et al. [24]	Bell et al. [25]	Faria et al. [26]	Abusin and Johnson [27]	Mainra and Card [28]	Zaman et al. [29]
Age (years)	30	45	57	38	2.2	47	44	24	38	14	17	9	33	22	24	23
Sex (M/F)	F	M	M	F	F	F	F	F	M	F	M	M	F	F	F	M
Ethnicity	Caucasian	Unknown	Asian	Unknown	Caucasian	African American	Unknown	Unknown	Unknown	Caucasian	Asian	African American	Unknown	Unknown	Unknown	Asian
Prescription reason	SIBO	UTI	Sinusitis	UTI	Recurrent Otitis Media	UTI	UTI	UTI	PJP Prophylaxis	PNA	Nodular cystic acne	MRSA skin and soft tissue infection of lower back and buttocks	UTI	UTI	Unkown	Otitis Externa
Systemic co‐morbid illnesses	Insomnia, Eczema	None	None listed	None listed	None listed	HTN, Iron Deficient Anemia, depression	None	None	ESRD s/p Renal transplant	CF	None	None	None	None listed	None	None
Concomitant medications	Neomycin (for SIBO)	Tamsulosin	None listed	None listed	None listed	Bupropion, venlafaxine, sumitriptan, losartan, HCTZ, iron tablets	Acetaminophen	None listed	Steroids, MMF, Tacrolimus, basiliximab, valganciclovir	Fluticasone‐salmeterol, dornase alpha aerosol, montelukast, pancrelipase	Isotretinoin	Acetaminophen	None	None listed	None listed	None
Concomitant supplements	Denied	Denied	None listed	None listed	None listed	Denied	None listed		None listed	Multivitamin	None	None listed	None listed	None listed	None listed	None listed
Amount of antibiotic until first symptoms (days)	5	5	14	5	14	5	4	1	3	1	—	11	—	2	—	7
Amount antibiotic received prior to presentation (days)	6.5	7	14	5	14	6	4	1	9	5	28	14	5	6	—	7
Days until admission	6.5	7	28	5	14	6	6	1	0	14	—	14	30	6	—	8
Presenting complaint	Body aches, fever, malaise	Scleral icterus & malaise	Scleral icterus, rash	Jaundice	Fever, lethargy	Chills, N/V, fevers & dark urine	Fever, epigastric pain, nausea, rash	Abdominal pain & nausea	Asymptomatic (LFT elevation)	Headache, fatigue, decerased oral intake, rash, angioedema	Fever, myalgias, erythematous maculopapular rash	Fever, headache, neck pain	Malaise, fatigue, anorexia, vomiting, fever, jaundice, dark urine, pruritic rash	Scleral icterus, N/V & malaise	Not listed	Bodyaches, fevers
*R* value at initial presentation^a^	76.1	47.8	0.8	—	3	0.5	16.9	130.8	3.5		22.3	12.2	0.8	95.3	—	20.1
Peak AST (U/L)	14214	2444	955	—	700	290	11055	5294	100	1347	2054	947	154	3077	—	11549
Peak ALT (U/L)	11373	3566	3303	—	650	175	6916	5233	400	1519	2862	624	103	4067	—	23289
Peak bili (mg/dL)	4.4	20.3	22.2	—	21.6	6.6	6.2	1	—	—	3.6	0.8	26.8	24.4	—	10.3
Peak INR	2.2	—	2	—	—	1.6	3.4	2	—	—	—	—	1.4	2.6	—	7.5
Encephalopathy present? (Y/N)	N	N	N	Unknown	Y	N	N	N	N	N	Y	Unknown	N	N	Unknown	Y
Complications	AKI	None listed	DRESS	None listed	Aspectic meningitis	None listed	None listed	None listed	None listed	None listed	DRESS	None listed	None listed	None listed	Erythema Multiforme and thrombocytopenia	None listed
Treatment received (if any)	NAC	None	Steroids	Ursodiol & steroids	Ursodiol	None	NAC	NAC	None listed	None listed	NAC, Vit K, molecular adsorbent recirculating system (MARS) extracorporeal liver dialysis	None listed	Cholestyramine and hydroxyzine	None	None listed	None listed
Outcome	Recovery	Recovery	Recovery	Transplant	Recovery	Recovery	Recovery	Recovery	Recovery	Recovery	Recovery	Unknown	Recovery	Recovery	Unknown	Transplant
Time to symptom improvement (days)	5	—	—	540	14	9	4	14	—	—	25	4	—	60	—	30
Time to lab normalization (days)	33	—	—	—	34	120	30	14	30	42	60	7	1050	60	240	180

^a^ *R*‐value surrogate for type of liver injury (Cholestatic vs. Hepatocellular vs. Mixed). Based upon the following equation: *R* = (ALT/ULN)/(ALP/ULN)^b^ ^,^ ^c^, where *R* > 5 indicates predominate hepatocellular, *R* < 2 indicates predominate cholestatic, 5 > *R* > 2 indicates mixed picture.

^b^ Standard ULN of ALT set at 40.

^c^ Standard ULN ALP set at 120.

First, this is the first case report to describe a TMP‐SMX DILI in the setting of treatment for SIBO. Although there is no direct evidence in the literature demonstrating an effect of SIBO on the absorption of TMP‐SMX, it is reasonable to hypothesize that SIBO could cause altered absorption of TMP‐SMX due to increased bacterial metabolism, drug sequestration, or changes in intestinal permeability and drug transporters, leading to liver injury [30].

A second highlight is the use of NAC in the treatment of TMP‐SMX DILI. As a review of recent literature demonstrates, NAC has only been used in three other cases where TMP‐SMX DILI was secondary to hepatocellular etiology [20, 21, 24]. In this case, clinical symptoms coupled with an *R*‐factor > 5 (*R* = 76.1 on admission), leukopenia, anemia, and coagulopathy strongly indicated a severe hepatocellular injury. Although current evidence on the impact of NAC on overall survival in TMP‐SMX DILI is inconclusive, data from other non‐acetaminophen DILI cases suggest that NAC may increase transplant‐free survival and shorten hospital stays [31]. There may also be a relationship between TMP‐SMX DILI and variants in human leukocyte antigens or N‐acetyltransferase genes [32, 33]. Given that NAC is well‐tolerated at standard doses, the favorable risk–benefit profile supports its use even when definitive evidence of efficacy is limited.

Third, this case is unique in that this patient had the second highest liver enzymes in recent literature. Yet, she did not experience hepatic encephalopathy and her liver imaging was unremarkable. Two other cases in the literature reported significantly lower transaminases, yet these patients developed encephalopathy [18, 24]. Comparatively, the case with closest transaminase elevation in the literature went on to require a liver transplant [29]. Calculation of a MELD score during admission (peak MELD of 20) prompted early implementation of specialist care and recommendation to utilize NAC early in her clinical course, which underscores the importance of early recognition of DILI and supportive treatment to prevent progression to acute liver failure [34].

## 6 Conclusion

This case report serves as a critical reminder that although many antibiotics are frequently utilized and generally considered safe, clinicians must maintain a high index of suspicion for DILI when patients develop constitutional symptoms. Medical professionals should remain vigilant when prescribing common agents such as TMP‐SMX, recognizing their potential for severe injury, ensuring prompt recognition of such injury, and acting swiftly to manage and prevent serious morbidity and mortality. The inherent diagnostic complexity that stems from the subtle and nonspecific presentation of DILI necessitates a comprehensive and systematic evaluation to exclude competing etiologies and establish diagnostic confidence. Because DILI can closely mimic viral, autoimmune, metabolic, or other intrinsic liver diseases, accurate diagnosis relies heavily on meticulous medication reconciliation and assessment of the temporal relationship between drug exposure and symptom onset. Furthermore, since patient reporting is often the primary catalyst for early detection, this case underscores the vital role of thorough patient education regarding the prodromal symptoms of hepatotoxicity. Such recognition, coupled with the observation of clinical and biochemical recovery following withdrawal of the suspected agent, remains the cornerstone of diagnosis, particularly in the absence of pathognomonic findings consistent with other etiologies. Following diagnostic confirmation, the clinical focus shifts to the mitigation of further injury through early initiation of supportive care. This discussion raises important considerations regarding therapeutic strategies in severe DILI. While evidence supporting specific pharmacologic interventions remains limited, the use of adjunctive therapies with favorable safety profiles may be justified in select high‐risk cases.

Collectively, this case contributes to the growing body of literature emphasizing the need for heightened clinical awareness of DILI, especially in the context of commonly used medications. Prompt recognition through patient education and comprehensive evaluation, timely initiation of supportive management, and cautious monitoring during treatment all play critical roles in preventing progression to acute liver failure and reducing the need for liver transplantation. From a research perspective, the findings advocate for improved standardization in case reporting and further exploration of pathophysiologic mechanisms and therapeutic interventions to enhance prevention, diagnosis, and management of drug‐induced liver injury.

## Author Contributions

**Kaylie Duit:** conceptualization, data curation, resources, writing – original draft writing, review and editing. **Joseph Rattenni:** conceptualization, data curation, resources, writing – original draft writing, review, and editing. **Lauryn Hanson:** data curation, resources, writing – original draft writing, review, and editing. **Aaron R. Kunz:** conceptualization, resources, supervision, writing – original draft writing, review, and editing.

## Funding

The authors have nothing to report.

## Consent

This statement serves to confirm that the authors obtained both verbal and written approval from the patient corresponding to the case report. Release of information paperwork was obtained to permit ongoing access to the patient's medical records. All details relevant to the clinical history were cross‐referenced with the patient for accuracy. The submitted draft of the manuscript was reviewed by the patient and had their approval for submission.

## Conflicts of Interest

The authors declare no conflicts of interest.

## Data Availability

The data that support the findings of this study are available from the corresponding author upon reasonable request.
